# Association of rs1800668 polymorphism in glutathione peroxidase- 1 gene and risk of rheumatoid arthritis in Pakistani population

**DOI:** 10.12669/pjms.325.10325

**Published:** 2016

**Authors:** Shazia Irfan, Asima Rani, Maryam Sameem, Syed Kashif Nawaz, Iram Liaqat, Muhammad Arshad

**Affiliations:** 1 Dr. Shazia Irfan, PhD Zoology. Department of Zoology, University of Sargodha, Sargodha, Pakistan; 2 Asima Rani, M.Phil Zoology. Department of Zoology, University of Sargodha, Sargodha, Pakistan; 3 Maryam Sameem, M.phil Zoology. Department of Zoology, University of Sargodha, Sargodha, Pakistan; 4 Dr. Syed Kashif Nawaz, PhD Zoology. Department of Zoology, University of Sargodha, Sargodha, Pakistan; 5 Dr. Iram Liaqat, PhD Zoology. Department of Zoology, GC University, Lahore, Pakistan; 6 Dr. Muhammad Arshad, PhD Zoology. University of Education, Lahore, Pakistan

**Keywords:** GPX1 C/T Polymorphism, Rheumatoid Arthritis, rs1800668

## Abstract

**Objective:**

To investigate the role of glutathione peroxidase 1 (GPX1) C/T polymorphism (rs1800668) in modulating the chances of Rheumatoid arthritis (RA) in Pakistani population.

**Methods:**

A total of 400 individuals including 200 controls and 200 patients of RA, were genotyped. Detection of rs1800668 polymorphism was carried out using PCR based amplification strategy (allele specific).

**Results:**

The results for Hardy Weinberg Equilibrium (HWE) indicated that the allele frequencies for GPX1 polymorphism were not deviant from HWE in whole population under observation. The statistical analysis indicated that significant association existed between rs1800668 polymorphism and RA (p<0.01). CT genotype increased the risk of RA development by 1.8582 times (OR: 1.8582; 95% CI 1.2154 to 2.8409). CC genotype was found to have protective effect against the disease development (OR: 0.5133; 95% CI 0.3403 to 0.7742) while TT genotype was found to have association with RA development but the risk level was marginal (OR: 1.5319; 95% CI 0.6124 to 3.8322).

**Conclusion:**

The present finding suggests the importance of GPX1 C/T polymorphism (rs1800668) in development of RA in Pakistani population. The protective role of CC genotype against the development of RA in local population was also observed.

## INTRODUCTION

Rheumatoid arthritis (RA) is systemic autoimmune disorder. It is an inflammatory form of arthritis which mainly affects the joints covered by synovial membrane. It results in swelling, pain and stiffness of joints and deformity. The exact cause of RA is still unknown. Many factors have been reported to contribute in the pathogenesis of RA including oxidative stress. Oxidative stress produces the reactive oxygen species (ROS) which generates the pro-inflammatory signals. Oxidative stress occurs either from the increased production of (ROS) or reactive nitrogen species (RNS) or due to problem in the antioxidant defense mechanisms.^1^,^2^

The cellular system controls the oxidative stress by several defense mechanisms, including intracellular enzymes. Most of the enzymes involved in the defense against oxidative stress are polymorphic. One of them is Glutathione Peroxidase (GPX). GPX is one of the most important antioxidant enzymes in humans body which helps in detoxification of hydrogen peroxide. There are five known forms of GPX: The form present in cells is GPX-1, in gastrointestinal is GPX-2, in plasma is GPX-3, in phospholipids is GPX-4 and in sperm is called snGPX.

The GPX1 enzyme is controlled by gene which is located at the chromosome 3 (p21 locus). The 5-UTR of GPX1 contains a single nucleotide C/T polymorphism (rs1800668) located at the position ch3:49395757. This polymorphism is important regarding activity of GPX1 enzyme. The CC genotype demonstrates relatively high activity of GPX1 as compared to CT or TT variant of alleles; however, the polymorphism does not alter the protein structure.^3^

The difference in enzymatic activity due to genotype disturbs the delicate balance between clearance of ROS and minimizing the oxidative stress. Reduced enzymatic activity and increased levels of ROS, such as superoxide radicals, occurs due to functional polymorphisms in antioxidant genes which play role in contribution towards the increased risk of age-related disorders.^4^

The GPX1 C/T polymorphism (rs1800668) is least studies polymorphism in relation to its association with different diseases. The role of this polymorphism has not been explored in RA disease. The objective of the current study was to analyze the role of GPX1 C/T polymorphism (rs1800668) as a risk factor or protective factor in the development of RA.

## METHODS

All procedures were in agreement with the declaration of Helsinki. The Advance Research and Study Board, University of Sargodha had approved the protocol of the present study. Ethical Committee, University of Sargodha granted permission for the start of research work. Written consent was also obtained from all patients. Study comprised of two groups named as RA group consisted of RA patients and the second control group which included age and sex matched healthy individuals. A form was designed to keep record of name, age, gender, smoking, hypertension, diabetes and RF factor status related to individuals.

The samples were collected during September 2013 to February 2014 from local hospitals. 200 RA patients (confirmed by RF test) and 200 healthy volunteers were selected for study. 3cc blood from each individual was collected in EDTA coated vials (BD, USA) for genetic analysis. Detection of rs1800668 polymorphism was carried out using PCR based amplification strategy (allele specific). GF-1 blood DNA extraction kit (Vivantis, USA) was used for Genomic DNA isolation. DNA was detected using 0.8% agarose gel while 2% agarose gel was used for resolving PCR product.

Primers were assembled by local representative of Invitrogen, USA. One reverse and two forward primers were used. Forward primer 1 (F1) 5’ CGCCTGCTGG CCTCCCCTTAC 3’Forward primer 2 (F2) 5’ CGCCTGCTGG CCTCCCCTTAT 3’and reverse primer (R)5’GCAGGGAGCCC AGGCTCACAG3’ were used to amplify rs1800668. Annealing temperature of primers was 62°C and PCR product size was 172 base pairs.

UV Transilluminator (BIOTOP Transilluminator, TU1002, China) was used to visualize genomic DNA and PCR products. In the presence of CC homozygous sample, bands (172bp) appear with F1 primer, in TT homozygous samples, bands (172bp) appear with F2 primer and in case of CT heterozygous samples, bands (172bp) appear with both F1 and F2 primers.

For analysis of Hardy Weinberg Equilibrium (HWE), gene frequencies, allele frequencies and difference in genetic and allelic frequencies Chi square test was used. Online calculator was used to calculate Odds ratio.^5^ Chi-square test and other non-parametric tests were applied by using SPSS Software version 16 for windows (SPAA Inc., Chicago Illinios, USA).

## RESULTS

The results of present study indicated no significant difference between RA and control group regarding age factor (Table-I).

**Table-I:** Baseline characteristics of RA patients and control group.

*Characteristics*	*RA patients (N = 200)*	*Control (N = 200)*	*Total (N = 400)*	*p-value*
Age (Years)	45.9± 10.4^a^	42.8± 10.6^a^	44.35±10.5^a^	0.74NS

a Data are shown as mean ± standard deviation.

Students T test was used for comparison of groups of RA and Control. S* = Significant (P<0.05); S** = Highly significant (P<0.01); NS = Non-significant (P>0.01)

Table-II describes the results for HWE estimation along with genotype and allele frequencies. The data indicates that T allelic frequency was low in control and RA group as compared to that of C allele. Allele frequencies were not deviant from HWE in control group as well as in RA patients.

**Table-II:** Genotype and allele frequencies of GPX1 rs1800668.

*Allele*	*Control (N=200)*	*RA (N=200)*	*Total (N=400)*
CC	140 (70%)	109 (54.5%)	249 (62.25%)
CT	52 (26%)	79 (39.5%)	131 (32.75%)
TT	08 (4%)	12 (6%)	20 (5%)
C	0.83	0.74	0.79
T	0.17	0.26	0.21
HWE (P)	1.24 (0.265)	0.22(0.639)	0.26(0.610)

Association was calculated in terms of Chi-square test (X^2^) and odds ratio with 95% confidence interval (95% CI). Table-III indicated that significant association existed between rs1800668 polymorphism and RA (p=0.00). CT genotype increased the risk of RA development by 1.8582 times (OR: 1.8582; 95% CI 1.2154 to 2.8409). CC genotype was found to have protective effect against the disease development (OR: 0.5133; 95% CI 0.3403 to 0.7742) while TT genotype was found to have association with RA development but risk level was marginal (OR: 1.5319; 95% CI 0.6124 to 3.8322).

**Table-III:** Association of genetic polymorphism in GPX1 rs1800668 and RA.

*GENOTYPE*	*RA*	*Control*	*ODDS RATIO*	*95%CI*	*Chi-square (p- value)*
CC	109	140	0.5133	0.3403 to 0.7742	10.22 (0.00 S**)
TT	12	08	1.5319	0.6124 to 3.8322
CT	79	52	1.8582	1.2154 to 2.8409

* S = Significant (P<0.05);

** S = Highly significant (P<0.01); NS = Non-significant (P>0.01).

Table-IV explains the association of RA with different risk factors which include smoking habit, diabetes and hypertension. Non-significant association existed between smoking and RA (p>0.01). Odds ratio test revealed that smoking increased the risk of RA but risk level was marginal (OD: 1.1945; 95% CI 0.5219 to 2.7339). Significant association of diabetes and hypertension with RA existed (p<0.01). Results suggested 2.6872 times increase in the risk of RA development in diabetic patients (OD 2.6872; CI 1.6811 to 4.2955). Odds ratio estimation describes that hypertension increased the risk of RA development by 13.0555 times (OD: 13.055; 95% CI 7.6566 to 22.2613).

**Table-IV:** Association of RA with different risk factors.

*Factors*	*Status*	*RA*	*Control*	*Odds ratio*	*95%CI*	*Chi-square (p-value)*
Smoking	Yes	13	11	1.1945	(0.5219-2.7339)	0.177 (0.673^NS^)
	No	187	189			
Diabetes	Yes	71	34	2.6872	1.6811-4.2955	17.679 (0.000 ^S**^)
	No	129	166			
Hypertension	Yes	121	21	13.0555	7.6566-22.2613	109.182 (0.00^S**^)
	No	79	179			

* S = Significant (P<0.05);

** S = Highly significant (P<0.01); NS = Non-significant (P>0.01)

## DISCUSSION

The etiology of RA is still partially understood but many studies have revealed the role of oxidative damage in RA which is critical for initiation and progression of RA.^6^ Multiple genetic variations have been associated with RA, but only a few have yet been confirmed. The diseases associated with elevated oxidative stress have been studied in relation to SNPs present in the antioxidant enzymes genes which may be useful in screening for diseases.^7^

The association between GPX1 C/T polymorphism (rs1800668) with risk of RA was analyzed in this study. We hypothesized that genetic variability in GPX1 gene could be involved in development of RA in Pakistani population.

To best of our knowledge, no studies have investigated the role of antioxidant gene GPX1 C/T polymorphism (rs1800668) for patients having RA. Only few studies has been conducted to analyze the association of GPX1 C>T polymorphism (rs1800668) with different diseases. A low risk association was found between GPX1 C>T polymorphism (rs1800668) with prostate cancer^8^ while marginal association existed between GPX1 rs1800668 C>T polymorphism and risk of prostate cancer.^9^ A study was carried out in Chinese Han population that provides no association between C/T polymorphism in *GPX-1*gene (rs1800668) and susceptibility to noise-induced hearing loss.^10^ Similarly numerous studies were conducted for risk of Alzheimer’s disease,^11^ for Kashin-Beck disease^12^ and risk of glioma, glioblastoma, meningioma, or acoustic neuroma for *GPX-1*C/T polymorphism (rs1800668)^13^ and found no association for this polymorphism.

Our observation is that CT genotype increased the risk of RA development, TT genotype was found to have association with RA development but risk level was marginal while CC genotype was found to have protective effect against the disease development. The protective effect of CC genotype might be due to that, this genotype demonstrates relatively high activity of GPX1 enzyme compared to CT or TT variant of alleles which is also reported in literature Najafi et al.^3^ in 2012. Our results suggest that polymorphism in GPX1 gene (rs1800668) which is involved in protection from oxidant stress is associated with RA.

There is a knowledge gap with respect to how GPX1 C/T polymorphism (rs1800668) in antioxidant gene may influence the development and pathogenesis of RA. Presently this research field has not been explored in detail and several questions related to it still exist. Present data can help in understanding the association of this polymorphism with RA disease but there is a need for further investigation in order to establish the true picture of association of GPX1 C/T polymorphism (rs1800668) with development of RA disease. Exact role of polymorphism will help to consider the disease associated polymorphisms as biomarkers in the disease diagnosis.

## CONCLUSION

The present finding suggests important role of rs1800668 in the development of RA in Pakistani population. CC genotype imparts protective effects towards the susceptibility of RA development.
